# Supplementary material for: Contextualising abortion opinions in Kenya: A vignette-based national survey
Source: PLOS Glob Public Health. 2026 Mar 3;6(3):e0006071. doi: 10.1371/journal.pgph.0006071 (PMC12956123; doi:10.1371/journal.pgph.0006071)
Supplement: S1 Table — (DOCX) [file pgph.0006071.s001.docx]

**S1** **Table. Model fit indices for situations in which participants would support latent classes 2–5**

| **NC** | **AIC** | **CAIC** | **BIC** | **Entropy** | **G^2^** |  |
| --- | --- | --- | --- | --- | --- | --- |
| 2 | 98,775.84 | 99,010.67 | 98,981.67 | 0.89 | 15,601.059 |  |
| 3 | 92,471.68 | 92,827.97 | 92,783.97 | 0.83 | 9,266.905 |  |
| 4 | 90,065.83 | 90,543.58 | 90,484.58 | 0.82 | 6,831.052 |  |
| 5 | 89,382.33 | 89,981.55 | 89,907.55 | 0.77 | 6,117.555 |  |
| **Note:** NC = Number of classes; AIC = Akaike's information criterion; CAIC = consistent Akaike information criterion; BIC = Bayesian information criterion; G^2^ = G square statistics; LMR LRT = Lo-Mendell-Rubin adjusted likelihood ratio test; *p-*value (A) = Bootstrap *p*-value for absolute model fit; *p*-value (B) = Bootstrap *p*-value for relative model fit | | | | | | |

We used the latent class analysis (LCA) method to identify underlying profiles of attitudes toward abortion, based on respondents’ levels of support across a range of hypothetical situations. LCA is a statistical technique that identifies unobserved (latent) subgroups within populations to characterise heterogeneity in response patterns (21). The LCA approach assumes that membership of a class is explainable by distinct, mutually exclusive patterns of observed variables across categorical sets of indicators (22).

We initially fitted a series of models specifying one to ten classes using poLCA (23) and a selected four-class solution based on the lowest Bayesian Information Criterion (BIC) and optimal entropy (0.82). To confirm model stability, we then estimated models with two to five classes using the glca package functions (24) in R version 4.3.1 (25). The latent classes were derived from 14 binary indicators representing situations in which participants indicated support for abortion. Based on model fit and interpretability, the four-class solution was retained. Missing data were handled using full information maximum likelihood estimation.
