# Supplementary material for: Contextualising abortion opinions in Kenya: A vignette-based national survey
Source: PLOS Glob Public Health. 2026 Mar 3;6(3):e0006071. doi: 10.1371/journal.pgph.0006071 (PMC12956123; doi:10.1371/journal.pgph.0006071)
Supplement: S1 Text — (DOCX) [file pgph.0006071.s009.docx]

**S1 Text. Questionnaire: Vignettes and accompanying questions and items**

| **1. FETAL ANOMALY** | | | | |
| --- | --- | --- | --- | --- |
| **Now, I am going to tell you a story. Please, listen to the story carefully. After the story, I will ask you a few questions.** | | | | |
| Wanjiku and her husband wanted a baby from the very beginning of their marriage because they felt lonely without a child. When they learned that Wanjiku was pregnant, they received the news with great joy. However, at 16 weeks, when the first ultrasound was performed, the doctor noticed that the foetus was anencephalic; that is, it had no brain. Upon hearing the diagnosis, Wanjiku’s first thought was to continue with the pregnancy. However, she also feared that keeping her pregnancy might cause even more suffering to her, her husband, and her foetus. Being in the second trimester of pregnancy, Wanjiku realised that the decision of whether to keep or interrupt the pregnancy would be extremely difficult.  Now keep this story about Wanjiku in mind as you answer the next few questions. | | | | |
| FA1 | Are you in favour of the right to abort in this situation? | Very favourable | ⃝ 1 | *Select one* |
|  |  | Favourable | ⃝ 2 |  |
|  |  | A little favourable | ⃝ 3 |  |
|  |  | A little contrary | ⃝ 4 |  |
|  |  | Contrary | ⃝ 5 |  |
|  |  | Very contrary | ⃝ 6 |  |
| FA2 | Are you in favour of the possibility of this type of abortion being performed in the Kenyan public health system? | Very favourable | ⃝ 1 | *Select one* |
|  |  | Favourable | ⃝ 2 |  |
|  |  | A little favourable | ⃝ 3 |  |
|  |  | A little contrary | ⃝ 4 |  |
|  |  | Contrary | ⃝ 5 |  |
|  |  | Very contrary | ⃝ 6 |  |
| FA3 | Would you do anything to support a woman to secure an abortion in this situation? | Certainly would NOT support | ⃝ 1 | *Select one* |
|  |  | Probably would NOT support | ⃝ 2 |  |
|  |  | Probably would support | ⃝ 3 |  |
|  |  | Certainly would support | ⃝ 4 |  |
|  |  | I don't know if I would support | ⃝ 5 |  |
| For the following statements, indicate your level of agreement. | | | |  |
| FA4 | Wanjiku and her husband have become responsible for a new life and must preserve it as long as possible. | I strongly agree | ⃝ 1 | *Select one* |
|  |  | I agree a little | ⃝ 2 |  |
|  |  | I am indifferent | ⃝ 3 |  |
|  |  | I disagree a little | ⃝ 4 |  |
|  |  | I strongly disagree | ⃝ 5 |  |
|  |  | I do not know | ⃝ 6 |  |
| FA5 | The protection of intrauterine life should be a duty of the Kenyan state even in cases of anencephaly. | I strongly agree | ⃝ 1 | *Select one* |
|  |  | I agree a little | ⃝ 2 |  |
|  |  | I am indifferent | ⃝ 3 |  |
|  |  | I disagree a little | ⃝ 4 |  |
|  |  | I strongly disagree | ⃝ 5 |  |
|  |  | I do not know | ⃝ 6 |  |
| FA6 | The foetus of Wanjiku cannot become a full human person because it has no prospects of psychologic development. | I strongly agree | ⃝ 1 | *Select one* |
|  |  | I agree a little | ⃝ 2 |  |
|  |  | I am indifferent | ⃝ 3 |  |
|  |  | I disagree a little | ⃝ 4 |  |
|  |  | I strongly disagree | ⃝ 5 |  |
|  |  | I do not know | ⃝ 6 |  |
| FA7 | A child should not be killed simply because nature did not give him the form his parents would like. | I strongly agree | ⃝ 1 | *Select one* |
|  |  | I agree a little | ⃝ 2 |  |
|  |  | I am indifferent | ⃝ 3 |  |
|  |  | I disagree a little | ⃝ 4 |  |
|  |  | I strongly disagree | ⃝ 5 |  |
|  |  | I do not know | ⃝ 6 |  |
| FA8 | Abortion should be based on a reliable diagnosis of anencephaly; that is, the certainty that the unborn child will never have a future. | I strongly agree | ⃝ 1 | *Select one* |
|  |  | I agree a little | ⃝ 2 |  |
|  |  | I am indifferent | ⃝ 3 |  |
|  |  | I disagree a little | ⃝ 4 |  |
|  |  | I strongly disagree | ⃝ 5 |  |
|  |  | I do not know | ⃝ 6 |  |
| FA9 | If the Supreme Court of Kenya allowed abortion in cases of anencephaly, it would make society less sensitive to the value of life. | I strongly agree | ⃝ 1 | *Select one* |
|  |  | I agree a little | ⃝ 2 |  |
|  |  | I am indifferent | ⃝ 3 |  |
|  |  | I disagree a little | ⃝ 4 |  |
|  |  | I strongly disagree | ⃝ 5 |  |
|  |  | I do not know | ⃝ 6 |  |
| FA10 | Forcing Wanjiku to maintain the pregnancy of an anencephalic foetus is the same as torturing her for the remainder of the pregnancy. | I strongly agree | ⃝ 1 | *Select one* |
|  |  | I agree a little | ⃝ 2 |  |
|  |  | I am indifferent | ⃝ 3 |  |
|  |  | I disagree a little | ⃝ 4 |  |
|  |  | I strongly disagree | ⃝ 5 |  |
|  |  | I do not know | ⃝ 6 |  |
| FA11 | Women who interrupt pregnancy in cases of anencephaly may be under pressure from their partners to do so, | I strongly agree | ⃝ 1 | *Select one* |
|  |  | I agree a little | ⃝ 2 |  |
|  |  | I am indifferent | ⃝ 3 |  |
|  |  | I disagree a little | ⃝ 4 |  |
|  |  | I strongly disagree | ⃝ 5 |  |
|  |  | I do not know | ⃝ 6 |  |
| FA12 | Interrupting a pregnancy in cases of anencephaly prevents the future suffering of the child if it is born. | I strongly agree | ⃝ 1 | *Select one* |
|  |  | I agree a little | ⃝ 2 |  |
|  |  | I am indifferent | ⃝ 3 |  |
|  |  | I disagree a little | ⃝ 4 |  |
|  |  | I strongly disagree | ⃝ 5 |  |
|  |  | I do not know | ⃝ 6 |  |
| FA13 | If Wanjiku comes to a definite position on what to do, then her will must be respected. | I strongly agree | ⃝ 1 | *Select one* |
|  |  | I agree a little | ⃝ 2 |  |
|  |  | I am indifferent | ⃝ 3 |  |
|  |  | I disagree a little | ⃝ 4 |  |
|  |  | I strongly disagree | ⃝ 5 |  |
|  |  | I do not know | ⃝ 6 |  |
|  |  | I agree a little | ⃝ 2 |  |
|  |  | I am indifferent | ⃝ 3 |  |
|  |  | I disagree a little | ⃝ 4 |  |
|  |  | I strongly disagree | ⃝ 5 |  |
|  |  | I do not know | ⃝ 6 |  |
| FA14 | Societies that respect the sexual and reproductive rights of their citizens allow abortion in cases of anencephaly. | I strongly agree | ⃝ 1 | *Select one* |
|  |  | I agree a little | ⃝ 2 |  |
|  |  | I am indifferent | ⃝ 3 |  |
|  |  | I disagree a little | ⃝ 4 |  |
|  |  | I strongly disagree | ⃝ 5 |  |
|  |  | I do not know | ⃝ 6 |  |
| FA15 | Interrupting pregnancy in cases such as this may mitigate the suffering of the mother and father of the anencephalic foetus. | I strongly agree | ⃝ 1 | *Select one* |
|  |  | I agree a little | ⃝ 2 |  |
|  |  | I am indifferent | ⃝ 3 |  |
|  |  | I disagree a little | ⃝ 4 |  |
|  |  | I strongly disagree | ⃝ 5 |  |
|  |  | I do not know | ⃝ 6 |  |
| FA16 | If she decides to interrupt the pregnancy, it is possible that Wanjiku will suffer from regret in the future. | I strongly agree | ⃝ 1 | *Select one* |
|  |  | I agree a little | ⃝ 2 |  |
|  |  | I am indifferent | ⃝ 3 |  |
|  |  | I disagree a little | ⃝ 4 |  |
|  |  | I strongly disagree | ⃝ 5 |  |
|  |  | I do not know | ⃝ 6 |  |
| **2. RAPE PREGNANCY** | | | | |
| **Now, I am going to tell you a story. Please listen to the story carefully. After the story, I will ask you a few questions.** | | | | |
| Zawadi is 23 years old, an evangelical Christian, and single. She was raped in her home by a gang of armed men. Because of fear, she did not seek immediate care, tell her parents, or report the incident to the police. She became pregnant as a consequence of the rape. When she realised she was pregnant, she felt at risk, frightened, and without options. At that moment, she sought care in a primary health unit, where she was advised of the legal possibilities of interrupting the pregnancy and what would be necessary if she decided to perform it. However, Zawadi remained confused. More than once, she was told that she was responsible for the pregnancy, because some did not believe that she had been raped while others felt she had put herself at risk of being raped. In addition, the induction of abortion contradicted her religious beliefs. Deeply distressed and without any support from friends or family, Zawadi does not know what to do. Now keep this story about Zawadi in mind as you answer the next few questions. | | | | |
| RP1 | Are you in favour of the right to abort in this situation? | Very favourable | ⃝ 1 | *Select one* |
|  |  | Favourable | ⃝ 2 |  |
|  |  | A little favourable | ⃝ 3 |  |
|  |  | A little contrary | ⃝ 4 |  |
|  |  | Contrary | ⃝ 5 |  |
|  |  | Very contrary | ⃝ 6 |  |
| RP2 | Are you in favour of the possibility of this type of abortion being performed by the Kenyan public health system? | Very favourable | ⃝ 1 | *Select one* |
|  |  | Favourable | ⃝ 2 |  |
|  |  | A little favourable | ⃝ 3 |  |
|  |  | A little contrary | ⃝ 4 |  |
|  |  | Contrary | ⃝ 5 |  |
|  |  | Very contrary | ⃝ 6 |  |
| RP3 | Would you do anything to support a woman to secure an abortion in that circumstance? | Certainly would NOT support | ⃝ 1 | *Select one* |
|  |  | Probably would NOT support | ⃝ 2 |  |
|  |  | Probably would support | ⃝ 3 |  |
|  |  | Certainly would support | ⃝ 4 |  |
|  |  | I don't know if I would support | ⃝ 5 |  |
| Tell me how much you agree with the statements below. | | | |  |
| RP4 | The right to abortion in rape cases is an important way to combat maternal mortality. | I strongly agree | ⃝ 1 | *Select one* |
|  |  | I agree a little | ⃝ 2 |  |
|  |  | I am indifferent | ⃝ 3 |  |
|  |  | I disagree a little | ⃝ 4 |  |
|  |  | I strongly disagree | ⃝ 5 |  |
|  |  | I do not know | ⃝ 6 |  |
| RP5 | Zawadi is now suffering because of rape, but may suffer even more if she aborts and feels guilty. | I strongly agree | ⃝ 1 | *Select one* |
|  |  | I agree a little | ⃝ 2 |  |
|  |  | I am indifferent | ⃝ 3 |  |
|  |  | I disagree a little | ⃝ 4 |  |
|  |  | I strongly disagree | ⃝ 5 |  |
|  |  | I do not know | ⃝ 6 |  |
| RP6 | The right to abortion in cases of sexual violence contravenes the constitutional principle of the defence of life. | I strongly agree | ⃝ 1 | *Select one* |
|  |  | I agree a little | ⃝ 2 |  |
|  |  | I am indifferent | ⃝ 3 |  |
|  |  | I disagree a little | ⃝ 4 |  |
|  |  | I strongly disagree | ⃝ 5 |  |
|  |  | I do not know | ⃝ 6 |  |
| RP7 | The unborn child should not be killed because Zawadi was unable to defend herself from a gang of armed men’s sexual aggression. | I strongly agree | ⃝ 1 | *Select one* |
|  |  | I agree a little | ⃝ 2 |  |
|  |  | I am indifferent | ⃝ 3 |  |
|  |  | I disagree a little | ⃝ 4 |  |
|  |  | I strongly disagree | ⃝ 5 |  |
|  |  | I do not know | ⃝ 6 |  |
| RP8 | In a secular state, the possibility of terminating pregnancy in cases of sexual violence is guaranteed. | I strongly agree | ⃝ 1 | *Select one* |
|  |  | I agree a little | ⃝ 2 |  |
|  |  | I am indifferent | ⃝ 3 |  |
|  |  | I disagree a little | ⃝ 4 |  |
|  |  | I strongly disagree | ⃝ 5 |  |
|  |  | I do not know | ⃝ 6 |  |
| RP9 | The right to termination of pregnancy in cases of violence mitigates the suffering of women. | I strongly agree | ⃝ 1 | *Select one* |
|  |  | I agree a little | ⃝ 2 |  |
|  |  | I am indifferent | ⃝ 3 |  |
|  |  | I disagree a little | ⃝ 4 |  |
|  |  | I strongly disagree | ⃝ 5 |  |
|  |  | I do not know | ⃝ 6 |  |
| RP10 | Zawadi should keep in mind that life is sacred regardless of how it was created. | I strongly agree | ⃝ 1 | *Select one* |
|  |  | I agree a little | ⃝ 2 |  |
|  |  | I am indifferent | ⃝ 3 |  |
|  |  | I disagree a little | ⃝ 4 |  |
|  |  | I strongly disagree | ⃝ 5 |  |
|  |  | I do not know | ⃝ 6 |  |
| RP11 | Legal abortion in cases of rape frees the victims of the horrors of carrying a life created in an act of terrible violence. | I strongly agree | ⃝ 1 | *Select one* |
|  |  | I agree a little | ⃝ 2 |  |
|  |  | I am indifferent | ⃝ 3 |  |
|  |  | I disagree a little | ⃝ 4 |  |
|  |  | I strongly disagree | ⃝ 5 |  |
|  |  | I do not know | ⃝ 6 |  |
| RP12 | If performed in the first weeks of gestation, abortion will affect an embryo, *even though* it does not yet have brain activity. | I strongly agree | ⃝ 1 | *Select one* |
|  |  | I agree a little | ⃝ 2 |  |
|  |  | I am indifferent | ⃝ 3 |  |
|  |  | I disagree a little | ⃝ 4 |  |
|  |  | I strongly disagree | ⃝ 5 |  |
|  |  | I do not know | ⃝ 6 |  |
| RP13 | Discontinuation of a healthy foetus pregnancy can be considered a form of violence against the weakest. | I strongly agree | ⃝ 1 | *Select one* |
|  |  | I agree a little | ⃝ 2 |  |
|  |  | I am indifferent | ⃝ 3 |  |
|  |  | I disagree a little | ⃝ 4 |  |
|  |  | I strongly disagree | ⃝ 5 |  |
|  |  | I do not know | ⃝ 6 |  |
| RP14 | Zawadi’s wish to have an abortion should be enough to allow her to safely interrupt the pregnancy. | I strongly agree | ⃝ 1 | *Select one* |
|  |  | I agree a little | ⃝ 2 |  |
|  |  | I am indifferent | ⃝ 3 |  |
|  |  | I disagree a little | ⃝ 4 |  |
|  |  | I strongly disagree | ⃝ 5 |  |
|  |  | I do not know | ⃝ 6 |  |
| RP15 | If Zawadi is lying, then she has no right to an abortion because she put herself in a situation to be raped. | I strongly agree | ⃝ 1 | *Select one* |
|  |  | I agree a little | ⃝ 2 |  |
|  |  | I am indifferent | ⃝ 3 |  |
|  |  | I disagree a little | ⃝ 4 |  |
|  |  | I strongly disagree | ⃝ 5 |  |
|  |  | I do not know | ⃝ 6 |  |
| RP16 | In hospitals, it is possible that many women lie to abort, trivialising the procedure. | I strongly agree | ⃝ 1 | *Select one* |
|  |  | I agree a little | ⃝ 2 |  |
|  |  | I am indifferent | ⃝ 3 |  |
|  |  | I disagree a little | ⃝ 4 |  |
|  |  | I strongly disagree | ⃝ 5 |  |
|  |  | I do not know | ⃝ 6 |  |
| RP17 | The legal possibility of abortion in cases of rape respects international agreements on sexual and reproductive human rights. | I strongly agree | ⃝ 1 | *Select one* |
|  |  | I agree a little | ⃝ 2 |  |
|  |  | I am indifferent | ⃝ 3 |  |
|  |  | I disagree a little | ⃝ 4 |  |
|  |  | I strongly disagree | ⃝ 5 |  |
|  |  | I do not know | ⃝ 6 |  |
| **3. RISK TO A WOMAN'S LIFE** | | | | |
| **I am going to tell you a story. Please listen to the story carefully. After the story, I will ask you a few questions.** | | | | |
| Pregnant for 13 weeks, Wawira is a carrier of the rare Eisenmenger’s Syndrome. The problem carries a high rate of maternal mortality. It causes the sufferer to experience extreme fatigue and breathlessness when performing even the simplest physical activities. The only cure is a heart transplant combined with a lung transplant. In the case of pregnancy, the medical indication is termination. Even abortion involves a risk for the pregnant woman, but it is lower than maintaining the pregnancy, especially if performed during the first trimester. However, Wawira and her husband desire very much to have a child. And, for the moment, the foetus is developing normally.  Now keep this story about Wawira in mind as you answer the next few questions. | | | | |
| RL1 | Are you in favour of the right to abort in this situation? | Very favourable | ⃝ 1 | *Select one* |
|  |  | Favourable | ⃝ 2 |  |
|  |  | A little favourable | ⃝ 3 |  |
|  |  | A little contrary | ⃝ 4 |  |
|  |  | Contrary | ⃝ 5 |  |
|  |  | Very contrary | ⃝ 6 |  |
| RL2 | Are you in favour of the possibility of this type of abortion being performed by the Kenyan public health system? | Very favourable | ⃝ 1 | *Select one* |
|  |  | Favourable | ⃝ 2 |  |
|  |  | A little favourable | ⃝ 3 |  |
|  |  | A little contrary | ⃝ 4 |  |
|  |  | Contrary | ⃝ 5 |  |
|  |  | Very contrary | ⃝ 6 |  |
| RL3 | Would you do anything to support a woman to secure an abortion in that circumstance? | Certainly would NOT support | ⃝ 1 | *Select one* |
|  |  | Probably would NOT support | ⃝ 2 |  |
|  |  | Probably would support | ⃝ 3 |  |
|  |  | Certainly would support | ⃝ 4 |  |
|  |  | I don't know if I would support | ⃝ 5 |  |
| Now tell me how much you agree with the statements below. | | | |  |
| RL4 | The maintenance of such a risky pregnancy would be the same as signing Wawira’s death certificate. | I strongly agree | ⃝ 1 | *Select one* |
|  |  | I agree a little | ⃝ 2 |  |
|  |  | I am indifferent | ⃝ 3 |  |
|  |  | I disagree a little | ⃝ 4 |  |
|  |  | I strongly disagree | ⃝ 5 |  |
|  |  | I do not know | ⃝ 6 |  |
| RL5 | The high mortality rate for pregnant women with this disease justifies abortion to protect the mother’s life. | I strongly agree | ⃝ 1 | *Select one* |
|  |  | I agree a little | ⃝ 2 |  |
|  |  | I am indifferent | ⃝ 3 |  |
|  |  | I disagree a little | ⃝ 4 |  |
|  |  | I strongly disagree | ⃝ 5 |  |
|  |  | I do not know | ⃝ 6 |  |
| RL6 | Recommending abortion in cases of Eisenmenger’s Syndrome is cruel because it protects only one of the two people involved. | I strongly agree | ⃝ 1 | *Select one* |
|  |  | I agree a little | ⃝ 2 |  |
|  |  | I am indifferent | ⃝ 3 |  |
|  |  | I disagree a little | ⃝ 4 |  |
|  |  | I strongly disagree | ⃝ 5 |  |
|  |  | I do not know | ⃝ 6 |  |
| RL7 | The legal possibility of abortion in such cases is an important way to combat maternal mortality. | I strongly agree | ⃝ 1 | *Select one* |
|  |  | I agree a little | ⃝ 2 |  |
|  |  | I am indifferent | ⃝ 3 |  |
|  |  | I disagree a little | ⃝ 4 |  |
|  |  | I strongly disagree | ⃝ 5 |  |
|  |  | I do not know | ⃝ 6 |  |
| RL8 | The laws that allow abortion in cases of Eisenmenger’s Syndrome fail to protect the unborn child. | I strongly agree | ⃝ 1 | *Select one* |
|  |  | I agree a little | ⃝ 2 |  |
|  |  | I am indifferent | ⃝ 3 |  |
|  |  | I disagree a little | ⃝ 4 |  |
|  |  | I strongly disagree | ⃝ 5 |  |
|  |  | I do not know | ⃝ 6 |  |
| RL9 | It is psychologically difficult for a healthcare professional to have to terminate the pregnancy of a healthy foetus. | I strongly agree | ⃝ 1 | *Select one* |
|  |  | I agree a little | ⃝ 2 |  |
|  |  | I am indifferent | ⃝ 3 |  |
|  |  | I disagree a little | ⃝ 4 |  |
|  |  | I strongly disagree | ⃝ 5 |  |
|  |  | I do not know | ⃝ 6 |  |
| RL10 | In countries where individual freedoms are respected, pregnant women like Wawira have the right to end the pregnancy if they wish. | I strongly agree | ⃝ 1 | *Select one* |
|  |  | I agree a little | ⃝ 2 |  |
|  |  | I am indifferent | ⃝ 3 |  |
|  |  | I disagree a little | ⃝ 4 |  |
|  |  | I strongly disagree | ⃝ 5 |  |
|  |  | I do not know | ⃝ 6 |  |
| RL11 | To accept abortion in such cases indicates lack of respect for the life of the unborn child. | I strongly agree | ⃝ 1 | *Select one* |
|  |  | I agree a little | ⃝ 2 |  |
|  |  | I am indifferent | ⃝ 3 |  |
|  |  | I disagree a little | ⃝ 4 |  |
|  |  | I strongly disagree | ⃝ 5 |  |
|  |  | I do not know | ⃝ 6 |  |
| RL12 | If Wawira decides to take the pregnancy forward, her attitude may be seen as heroism by her family. | I strongly agree | ⃝ 1 | *Select one* |
|  |  | I agree a little | ⃝ 2 |  |
|  |  | I am indifferent | ⃝ 3 |  |
|  |  | I disagree a little | ⃝ 4 |  |
|  |  | I strongly disagree | ⃝ 5 |  |
|  |  | I do not know | ⃝ 6 |  |
| RL13 | At 13 weeks, Wawira’s foetus has a lower capacity to feel pain than a born person. | I strongly agree | ⃝ 1 | *Select one* |
|  |  | I agree a little | ⃝ 2 |  |
|  |  | I am indifferent | ⃝ 3 |  |
|  |  | I disagree a little | ⃝ 4 |  |
|  |  | I strongly disagree | ⃝ 5 |  |
|  |  | I do not know | ⃝ 6 |  |
| RL14 | Wawira must have complete freedom to choose to abort or maintain the pregnancy because it is her life and her body that are at risk. | I strongly agree | ⃝ 1 | *Select one* |
|  |  | I agree a little | ⃝ 2 |  |
|  |  | I am indifferent | ⃝ 3 |  |
|  |  | I disagree a little | ⃝ 4 |  |
|  |  | I strongly disagree | ⃝ 5 |  |
|  |  | I do not know | ⃝ 6 |  |
| RL15 | Being able to interrupt the pregnancy in case of risk of death for the pregnant woman is a fundamental right. | I strongly agree | ⃝ 1 | *Select one* |
|  |  | I agree a little | ⃝ 2 |  |
|  |  | I am indifferent | ⃝ 3 |  |
|  |  | I disagree a little | ⃝ 4 |  |
|  |  | I strongly disagree | ⃝ 5 |  |
|  |  | I do not know | ⃝ 6 |  |
| RL16 | Performing abortion in cases like this affects a foetus that could have a normal future ahead of it. | I strongly agree | ⃝ 1 | *Select one* |
|  |  | I agree a little | ⃝ 2 |  |
|  |  | I am indifferent | ⃝ 3 |  |
|  |  | I disagree a little | ⃝ 4 |  |
|  |  | I strongly disagree | ⃝ 5 |  |
|  |  | I do not know | ⃝ 6 |  |
| RL17 | Wawira and her husband were aware of the risks when they freely decided to fulfil the dream of having a child. | I strongly agree | ⃝ 1 | *Select one* |
|  |  | I agree a little | ⃝ 2 |  |
|  |  | I am indifferent | ⃝ 3 |  |
|  |  | I disagree a little | ⃝ 4 |  |
|  |  | I strongly disagree | ⃝ 5 |  |
|  |  | I do not know | ⃝ 6 |  |
