# Supplementary material for: Contextualising abortion opinions in Kenya: A vignette-based national survey
Source: PLOS Glob Public Health. 2026 Mar 3;6(3):e0006071. doi: 10.1371/journal.pgph.0006071 (PMC12956123; doi:10.1371/journal.pgph.0006071)
Supplement: Attachment — Submitted filename: Response to Reviewers.docx [file pgph.0006071.s010.docx]

The Editor,

PLOS Global Public Health

Dear Editor,

**Rebuttal letter to editor and reviewer comments (PGPH-D-25-01893)**

We are pleased to submit our revised manuscript following careful consideration of the editor's and reviewers' recommendations for revision. In this response letter, we have provided a point-by-point response to the editor and reviewers, specifying changes where necessary and answering reviewer questions when no actual change in the manuscript was warranted.

We are grateful to the editor and the two reviewers for their time and suggestions to improve the manuscript. We accept the revise-and-resubmit decision and have addressed all points raised, as outlined below.

EDITOR REQUESTS AND COMMENTS

Please include the following items when submitting your revised manuscript:

- A rebuttal letter that responds to each point raised by the editor and reviewer(s). You should upload this letter as a separate file labeled 'Response to Reviewers'. This has been provided (this document).
- A marked-up copy of your manuscript that highlights changes made to the original version. You should upload this as a separate file labeled 'Revised Manuscript with Track Changes'. A marked-up copy of the manuscript has been created and uploaded.
- An unmarked version of your revised paper without tracked changes. You should upload this as a separate file labeled 'Manuscript'. An unmarked version has been created and uploaded.

JOURNAL REQUIREMENTS

1. In the ethics statement in the Methods, you have specified that verbal consent was obtained. Please provide additional details regarding how this consent was documented and witnessed, and state whether this was approved by the IRB

Response: We have addressed this requirement. We have added text to indicate that permission to use verbal consent was requested in advance and granted by the IRB. We have also described how consent was recorded and witnessed.

2. Please amend your detailed Financial Disclosure statement. This is published with the article. It must therefore be completed in full sentences and contain the exact wording you wish to be published.

Response: The financial disclosure statement has been revised to indicate the funder (Sida) and grant number (12103), which was awarded to APHRC, where all the authors worked and were supported by the grant.

3. Please clarify all sources of financial support for your study. List the grants, grant numbers, and organizations that funded your study, including funding received from your institution. Please note that suppliers of material support, including research materials, should be recognized in the Acknowledgements section rather than in the Financial Disclosure.

Response: All aspects of the study were supported by one grant from Sida (contribution number 12103).

4. State the initials, alongside each funding source, of each author to receive each grant. For example: "This work was supported by the National Institutes of Health (####### to AM; ###### to CJ) and the National Science Foundation (###### to AM)."

Response: This has been revised to read, "This work was funded by Sida (12103 to APHRC and supported BAU, IGA, EM, and KJ)”.

5. State what role the funders took in the study. If the funders had no role in your study, please state: “The funders had no role in study design, data collection and analysis, decision to publish, or preparation of the manuscript.”

Response: As the funders played no role, we have adopted the recommended statement: “The funders had no role in study design, data collection and analysis, decision to publish, or preparation of the manuscript.”

6. If any authors received a salary from any of your funders, please state which authors and which funders.

Response: The authors did not receive salaries from the funders; they were paid their normal salaries as APHRC staff.

7. In the online submission form, you indicated that All data and materials are available on reasonable request from the corresponding author. Also, according to the APHRC policies (the organization hosting the datasets), all deidentified datasets will be publicly available on the APHRC microdata portal after three years (<https://aphrc.org/microdata-portal/>).

All PLOS journals now require all data underlying the findings described in their manuscript to be freely available to other researchers, either
1. In a public repository,
2. Within the manuscript itself, or
3. Uploaded as supplementary information.

This policy applies to all data except where public deposition would breach compliance with the protocol approved by your research ethics board. If your data cannot be made publicly available for ethical or legal reasons (e.g., public availability would compromise patient privacy), please explain your reasons by return email and your exemption request will be escalated to the editor for approval. Your exemption request will be handled independently and will not hold up the peer review process, but will need to be resolved should your manuscript be accepted for publication. One of the Editorial team will then be in touch if there are any issues.

Response: We have modified our statement on data availability, as we have now made all data available within the manuscript: All data underlying the findings described in this manuscript can be accessed via 10.6084/m9.figshare.30719501.

8. Please provide separate figure files in .tif or .eps format.

Response: We have recreated our files in .tif and uploaded them as separate files.

9. We have noticed that you have uploaded Supporting Information files, but you have not included a list of legends. Please add a full list of legends for your Supporting Information files after the references list.

Response: We have implemented this recommendation by creating a legend listing all the supporting files.

10. If the reviewer comments include a recommendation to cite specific previously published works, please review and evaluate these publications to determine whether they are relevant and should be cited. There is no requirement to cite these works unless the editor has indicated otherwise.

Response: While one reviewer suggested additional literature and citation, no specific published work was suggested.

11. Please review your reference list to ensure that it is complete and correct. If you have cited papers that have been retracted, please include the rationale for doing so in the manuscript text, or remove these references and replace them with relevant current references. Any changes to the reference list should be mentioned in the rebuttal letter that accompanies your revised manuscript. If you need to cite a retracted article, indicate the article’s retracted status in the References list and also include a citation and full reference for the retraction notice.

Response: The authors have carefully reviewed the reference list and ensured all cited materials are listed.

**REVIEWER COMMENTS**

REVIEWER #1

1. This is a well written paper, and the Introduction suitably makes the case to study public opinions on abortion in Kenya in the context of rape, threat to health and life, and fetal anomalies.

Response: We thank this reviewer for such kind comments.

2. Regarding the study design, it is stated that the database used is maintained by GeoPoll, a mobile phone-based survey provider and that consists of over 12 million contacts at the time of the survey. Do we assume that all service provider customers are included?

Response: Yes, mobile phone users of all telephonic service providers are included in the database. GeoPoll is not a mobile phone service provider, but it has amassed mobile phone users in its database as part of its mobile survey infrastructure, including users of diverse service providers.

3. The authors should be consistent in the spelling of GeoPoll. Elsewhere it is spelt Geopoll (without a capital ‘P’).

Response: Thank you very much for this comment. We confirm that the official name is GeoPoll. Therefore, we have corrected all mentions to ensure the name is accurate and used consistently throughout.

4. There were 21.3% that did not meet the inclusion criteria, and 5% (6,140) declined to participate. Are their demographics identified and are they very different from those that participated?

Response: Thank you for this critical question. We did not collect detailed information about those who refused to participate or who were ineligible. Once ineligibility or refusal was established, the interaction ended. In hindsight, terminating the interaction at that was a missed opportunity. However, it would also have been untenable to collect such information from potential participants who declined or were ineligible.

5. Table 1 aptly summarises the ‘Constructs of the moral reasoning related to abortion’. The reader subsequently has to constantly refer to that table as to what the abbreviations are. However, in Table 6 (Mean summary of scores from the mosaic of opinions on induced abortion constructs) there is another listing of the constructs and their abbreviations. The reviewer wonders if it may make the article too wordy to state the construct in full, though it is quite conventional to have abbreviations after first defining them.

Response: We felt that defining the constructs with their acronyms, as done in Table 1 and subsequently using abbreviations was more suitable to reduce cluttering and wordiness. We have decided to follow the standard pattern of using abbreviations after they have been defined in full. As such, Table 1 and Table 6 now list only the abbreviations, not both the abbreviations and the full names, having already defined them in the paragraph preceding Table 1.

6. Note the error in line 166 referring to the reference for Table 1. It is not From Cacique et al (2028), but From Cacique et al (2018). Also, why not place the citation number which is (14).

Response: Thank you for pointing out this error. It has been corrected. We have also used the citation number for consistency throughout the document.

7. Noted the results summarised in Tables 3 to 5 (Pearson’s chi squared test) and the reader looks forward to the multivariate analysis which follows. In the narrative related to Tables 7 and 8, it may be useful to use one example from either construct/variable to relate the numbers to the (already stated) narrative in the context of multivariate analysis. Does the Discussion address the significant variables (if any)?

Response: Thank you for this suggestion. We have implemented it. Regarding whether the discussion addresses significant variables, we have reviewed it and confirmed that it does.

8. The Supplementary material is very informative, and the authors could do well to lead the reader onto them explicitly in the text.

Response: Thank you. The authors appreciate the reviewer’s suggestion to lead readers more explicitly to the Supplementary Materials. We have added explicit pointers to Supplementary Table S1 and Supplementary Figures S1–S7 throughout the Results and Figure 1 legend. Specifically, we (1) cite Supplementary Table S1 and report the entropy used to justify the selected four-class LCA solution; (2) direct readers to Supplementary Figure S1 for bootstrapped mean construct scores and confidence intervals that underpin the class summaries; and (3) direct readers to Supplementary Figures S2–S7 for the vignette-specific item-level profiles that support our class interpretations.

9. Noteworthy that Figures S2 to S7 show for the various attitudes (statements 1 to 7 and 8 to 14) were overwhelmingly either ‘You agree too much’ or ‘You agree too little’ with few hedging and even fewer neutral.

Response: Yes, this is true. We have addressed this in the new paragraph we added to introduce the supplemental materials in the text.

10. For line 347 ......For example, (3) identified a group of respondents classified as “dilemma people”. Suggest ..... For example, Rye and Underhill (year), identified a group of respondents classified as “dilemma people”......... (3).

Response: Done. Thank you very much.

11. Similarly, line 357 ......Further support for this finding is a study by (7), which found that people can simultaneously..... Suggest, Further support for this finding is a study by (authors), which found that people can simultaneously..... (7).

Response: Done. Thank you very much.

12. The study limitations are well noted. Nevertheless, even without generalising to the whole Kenyan population, rich data has been captured and presented from the sample that though may have been incomplete (of all phone users) was a representaive sample (of the GeoPoll database).

Response: Thank you for this supportive comment.

13. Notwithstanding the limitations, the vignettes allow policy makers and other stakeholders an insight into a number of types of moral constructs categorised as liberal or conservative related to the scenarios related to abortion in the Kenyan context. Other countries with similar legislation would likely have similar public opinions.

Response: This is an accurate assessment and concluding statement following the limitation section. We have adopted and included it in the manuscript.

REVIEWER #2

1. The methodology adopted in this study is commendably detailed and systematic. Nevertheless, the review of relevant literature appears limited. Considering the growing body of recent research on abortion surveys, the inclusion of additional scholarly works would enhance the robustness of the methodological framework and provide stronger empirical support for the study’s findings.

Response: The authors thank reviewer #2 for this feedback. We have added text to the introduction, along with additional literature to situate the study and justify the findings in the discussion.

FIGURE RESUBMISSIONS:

1. While revising your submission, we strongly recommend that you use PLOS’s NAAS tool (<https://ngplosjournals.pagemajik.ai/artanalysis>) to test your figure files. NAAS can convert your figure files to the TIFF file type and meet basic requirements (such as print size, resolution), or provide you with a report on issues that do not meet our requirements and that NAAS cannot fix. After uploading your figures to PLOS’s NAAS tool - <https://ngplosjournals.pagemajik.ai/artanalysis>, NAAS will process the files provided and display the results in the "Uploaded Files" section of the page as the processing is complete. If the uploaded figures meet our requirements (or NAAS is able to fix the files to meet our requirements), the figure will be marked as "fixed" above. If NAAS is unable to fix the files, a red "failed" label will appear above. When NAAS has confirmed that the figure files meet our requirements, please download the file via the download option, and include these NAAS processed figure files when submitting your revised manuscript.

Response: We have prepared the figures and checked them all through PLOS’s NAAS tool. The uploaded files comply with the requirements for figures.

Thank you very much for your support. We look forward to getting a final decision on our manuscript.

Sincerely,

Boniface Ushie

For All co-authors
