# Supplementary material for: Contextualising abortion opinions in Kenya: A vignette-based national survey
Source: PLOS Glob Public Health. 2026 Mar 3;6(3):e0006071. doi: 10.1371/journal.pgph.0006071 (PMC12956123; doi:10.1371/journal.pgph.0006071)
Supplement: Attachment — Submitted filename: Response_to_Reviewers_auresp_2.docx [file pgph.0006071.s011.docx]

The Editor,

PLOS Global Public Health

Dear Editor,

**Rebuttal letter to editor and reviewer comments (PGPH-D-25-01893)**

We appreciate the editor’s support.

We accept the minor suggestions to improve the manuscript and have revised it as outlined below.

1. Please upload a file showing your changes either highlighted or using a track changes function in your word processor. This document should be uploaded as the item type 'Revised Article with Changes Highlighted.'

Response: We have now labelled the revised manuscript with tracked changes as “Revised Article with Tracked Changes”. We initially uploaded it as “Supplemental Information” per the instructions.

Thank you very much for your support. We look forward to the publication of this manuscript.

Sincerely,

Boniface Ushie

For all co-authors
